# Scorbutic Dysentery

**Published:** 1870-10

**Authors:** J. E. Thornburgh

**Affiliations:** Lynnville, Indiana


ARTICLE IV.—*Scorbutic Dysentery.* By J. E. THORNBURGH, M.D., Lynnville, Indiana.

Isaac L., aged nineteen years, American, was attacked July, 1869, with acute dysentery, which terminated finally in chronic dysentery. Previous to this attack, his habits had not been regular. He was intemperate, and drank great quantities of inferior whisky. He used a great amount of tobacco.

Saccharine articles were his principal diet. His digestion had not been good for several years, and he had had numerous attacks of acute dyspepsia. Several physicians had treated him for the chronic dysentery, all unsuccessfully. He came under my observation in March, 1870. He was extremely emaciated, with a marked sallowness of skin and slight œdema of lower extremities. His gums were in a terrible condition, bleeding profusely almost every day, very spongy, and teeth loose. The breath was very fetid. Ecchymoses numerous on lower extremities, chiefly in the form of petechiæ. The urine was very highly colored, having the appearance of containing blood. On examination none was found, but there was great abundance of the urates.

The discharges from the bowels were peculiarly offensive, and contained an abundance of mucus, and occasionally a small quantity of blood. Ulcerations extended to the extreme outer edge of the anus, causing severe pain on defecation.

Physical examination elicited slight tenderness over the greater part of the abdomen. The liver was very much atrophied and irregular.

March 12th, I placed him under the treatment usually employed in scorbutics, with wine. On the 14th of same month a profuse hemorrhage from the gums occurred, which astringents would not arrest. The Per Sulphate of Iron would arrest it for a few minutes only. I gave him some lemon juice to rinse his mouth with, and the hemorrhage immediately stopped. No more severe hemorrhages occurred after that time. The scorbutic symptoms gradually disappeared, with the exception of pains in his lower extremities, which still persisted and were sometimes very severe.

The treatment for the chronic dysentery was not so successful. Sub-nitrate of Bismuth, combined with Nitrate of Silver, appeared to give more benefit than any other remedies. The discharges became less frequent and more natural under the above remedies, and the patient recovered gradually, till the 18th of May, when he became suddenly worse. The discharges became very frequent, and were mixed with blood and pus.

May 20th, the patient became comatose, and, on the morning of the 21st, died.

There were no positive symptoms of peritonitis.

Was the diagnosis of scorbutic dysentery correct? If so, what caused the scorbutic element in the disease? What caused the atrophy of the liver?

